# Does Physical Inactivity Induce Significant Changes in Human Gut Microbiota? New Answers Using the Dry Immersion Hypoactivity Model

**DOI:** 10.3390/nu13113865

**Published:** 2021-10-29

**Authors:** Maxence Jollet, Kevin Nay, Angèle Chopard, Marie-Pierre Bareille, Arnaud Beck, Vincent Ollendorff, Barbara Vernus, Anne Bonnieu, Mahendra Mariadassou, Olivier Rué, Frédéric Derbré, Bénédicte Goustard, Christelle Koechlin-Ramonatxo

**Affiliations:** 1 DMEM, Université de Montpellier, INRAE, 34000 Montpellier, France; maxence.jollet@inra.fr (M.J.); Kevin.Nay@acu.edu.au (K.N.); angele.chopard@umontpellier.fr (A.C.); vincent.ollendorff@inrae.fr (V.O.); barbara.vernus@inrae.fr (B.V.); anne.bonnieu@inrae.fr (A.B.); benedicte.goustard@inrae.fr (B.G.); 2 Laboratoire “Movement Sport and Health Sciences”, University of Rennes/ENS Rennes, 35000 Rennes, France; frederic.derbre@univ-rennes2.fr; 3 Exercise and Nutrition Research Program, Mary MacKillop Institute for Health Research, Australian Catholic University, Melbourne, VIC 3000, Australia; 4 Institut de Médicine et Physiologie Spatiales (MEDES), 31400 Toulouse, France; marie-pierre.bareille@medes.fr (M.-P.B.); arnaud.beck@medes.fr (A.B.); 5 INRAE, BioinfOmics, MIGALE Bioinformatics Facility, Université Paris-Saclay, 78350 Jouy-en-Josas, France; mahendra.mariadassou@inrae.fr (M.M.); olivier.rue@inrae.fr (O.R.); 6 INRAE, MaIAGE, Université Paris-Saclay, 78350 Jouy-en-Josas, France

**Keywords:** hypoactivity, commensal bacteria, flora, phyla, muscle atrophy, disuse, weightlessness, micro-gravity

## Abstract

Gut microbiota, a major contributor to human health, is influenced by physical activity and diet, and displays a functional cross-talk with skeletal muscle. Conversely, few data are available on the impact of hypoactivity, although sedentary lifestyles are widespread and associated with negative health and socio-economic impacts. The study aim was to determine the effect of Dry Immersion (DI), a severe hypoactivity model, on the human gut microbiota composition. Stool samples were collected from 14 healthy men before and after 5 days of DI to determine the gut microbiota taxonomic profiles by 16S metagenomic sequencing in strictly controlled dietary conditions. The α and β diversities indices were unchanged. However, the operational taxonomic units associated with the Clostridiales order and the *Lachnospiraceae* family, belonging to the Firmicutes phylum, were significantly increased after DI. Propionate, a short-chain fatty acid metabolized by skeletal muscle, was significantly reduced in post-DI stool samples. The finding that intestine bacteria are sensitive to hypoactivity raises questions about their impact and role in chronic sedentary lifestyles.

## 1. Introduction

The human intestine houses 100 trillion bacteria, referred to as gut microbiota. These rich and diverse bacterial communities live in symbiosis with the host and play a key role in human health [1]. The gut microbiota composition is influenced by various factors, such as birth mode, living environment, diet, and antibiotic intake. Several studies have described links also with physical activity and energy expenditure, and recent reviews have highlighted the reciprocal interactions between physical activity and gut microbiota [1,2,3,4,5]. For instance, athletes display a specific gut microbiota composition. However, besides the intense physical activity, the dietary intake patterns of athletes are different from those of sedentary subjects [6] and this also might influence their gut microbiota composition [7,8]. Nevertheless, intervention studies with different training modalities in healthy sedentary or diseased populations (age-related pathologies, gastrointestinal diseases, metabolic or inflammatory diseases as obesity or osteoarthritis…) support the beneficial impact of exercise and physical activity on the gut microbiota [3,9,10].

The gut microbiota, as a modulator of the immune system, contributes to the intestinal homeostasis and gut integrity (permeability and inflammation) and also exerts a nutritional role through the production of vitamins and short chain fatty acids (SCFAs, used as fuel for epithelial cells and to regulate gene expression) and the regulation of lipid metabolism and low-grade inflammation [11,12]. In addition, our laboratory revealed a functional cross-talk between gut microbiota and skeletal muscle, a tissue with essential roles in energy balance regulation, body weight composition and physical performance. Specifically, we demonstrated in mice that gut microbiota depletion (by antibiotic treatment) affects the intrinsic contractile muscle endurance capacity and glucose homeostasis. These deleterious effects were normalized by reseeding with natural bacteria [13]. Studies using germ-free animal models or probiotics as a potential ergogenic aid to enhance physical performance [3,14] further support the hypothesis that targeted gut microbiota modulation is essential for muscle function and physical performance. All these findings pave the way for the development of therapeutic tools to manipulate the gut microbiota with the aim of optimizing (in athletes) or restoring muscle function (in patients with diseases that impair skeletal muscle, such as myopathies, cachexia, sarcopenia, or with “deconditioned” muscles due to hypoactivity).

On the other hand, the effects of hypoactivity on the human gut microbiota remain largely understudied. Limited data, derived from space medicine, suggest that gut bacteria are sensitive to microgravity because spaceflight affects the microbial composition of the astronauts’ gastrointestinal tract [15,16,17]. Currently, hypoactivity and sedentary lifestyles are widespread and have negative health and socio-economic consequences [18]. Epidemiological surveys by the World Health Organization indicate that in the world, 31% of the ≥15-year-old population has sedentary behavior and that sedentary lifestyles represent the fourth strongest risk factor of death in the world (6%). Indeed, lack of physical activity promotes obesity, cardiovascular diseases, type II diabetes, cancer, and skeletal muscle weakness. Therefore, the scientific and medical community should study the potential functional links between hypoactivity and gut microbiota composition, a major determinant of the host’s health.

The aim of this interventional study was to determine the effect of Dry Immersion (DI), an innovative severe hypoactivity model, on the gut microbiota composition of healthy men in strictly controlled dietary conditions. Analysis of the 16S rRNA metagenomic sequencing data allowed for determining the α and β diversity and the abundance of phyla, orders and families in stool samples collected before and after five days of DI. Our findings indicate that a short but severe physical inactivity period was enough to induce muscle atrophy and to cause several changes at the lower taxonomy levels and to the availability of microbe-derived propionate, whereas the gut microbiome global composition was preserved. As skeletal muscle, intestinal bacteria are sensitive to hypoactivity, raising questions on their mutual impacts and roles in chronic sedentary lifestyles.

## 2. Materials and Methods

### 2.1. Dry Immersion

To investigate the effects of physical inactivity on the human gut microbiota composition, the DI approach, previously described, was used [19]. Briefly, participants are loosely enveloped in elastic waterproof tarpaulin and then immersed in thermally neutral water. Therefore, they remain dry, hence the term of DI. DI accurately reproduces the effects of inactivity [20] quicker than the head-down bedrest model. Moreover, in this approach, supporting structures for the body are lacking. Recent studies that compared the cardiovascular, postural and neuromuscular changes after 21 days of bedrest and 3 days of DI reported similar effects (in amplitude), suggesting that DI induces much faster physiological changes due to weightlessness [21]. For this study, participants underwent DI for 5 days. In one group of participants, venoconstrictive thigh cuffs also were used, a countermeasure to sequester fluids in the lower limbs.

### 2.2. Participants

Twenty healthy men were recruited for this study, but two participants were excluded, for reasons unrelated to the protocol, four days before DI initiation (BDC-4). At BDC-2 (2 days before DI initiation), the remaining 18 men were randomly divided into the Control (*n* = 9) and Cuffs (*n* = 9) groups. Participants were anonymized and designated in data sets by single letters: B, E, F, I, K, M, O, Q and S were in the Control group, and A, C, D, G, H, J, N, P and R were in the Cuffs group. All participants were informed about the experimental procedures and signed written informed consent. The experimental protocol followed the standards set by the Declaration of Helsinki and was approved by the local Ethics Committee (CPP Est III: October 2, 2018, ID number RCB 2018-A01470-55) and French Health Authorities (ANSM: August 13, 2018; ClinicalTrials.gov Identifier: NCT03915457). Comparison (unpaired *t*-test) of the baseline characteristics of the two groups (Table 1) did not highlight any significant difference.

### 2.3. Body Composition and Diet

Fat and lean free mass were measured by dual-energy X-ray absorptiometry (DEXA) (Hologic, QDR 4500 C, Bedford, MA, USA) 4 days before DI (BDC-4), and after 5 days of DI (DI-5). The menu composition was identical for all participants from BDC-4 to day 2 of ambulatory recovery (R0, R + 1). Dietary intake was individually tailored and controlled throughout the study. The intake of carbohydrates (CHO), proteins, fatty acids, total water, fibers, minerals and vitamins was recorded each day from DI-4 to R + 1. The adequate water intake was fixed at 35–60 mL/kg/day; within this range water intake throughout the protocol was ad libitum (measured). The individual energy intake was calculated by multiplying the resting metabolic rate by the physical activity levels before and during DI (1.6 and 1.3, respectively).

### 2.4. Overall Study Design

The study was carried out at the MEDES space clinic, Toulouse, France from November 19, 2018 to March 23, 2019. Participants arrived at BDC-5 in the evening and left at R + 2 (recovery day 3) in the morning. The experimental protocol included four days of ambulatory baseline measurements before DI (BDC-4 to BDC-1), five days (120 h) of DI (DI-1 to DI-5), and two days of ambulatory recovery (R0, R + 1). In addition, one week before the protocol initiation, participants went to the MEDES clinic for resting metabolic rate measurement. DI was performed according to the methodology detailed in [22]. Participants were paired (*n* = 1 from the Control group and *n* = 1 from the Cuffs group). Each pair underwent DI simultaneously in the same room, in two separate baths (except for one Cuffs and one Control participant, C and M, who had no mate). The water temperature was continuously maintained in the thermal neutral zone. The light-off period was set at 23:00–07:00. Daily hygiene, weighing, and some specific measurements required extraction from the bath. During these out-of-bath periods, participants were in the −6° head-down position, a reliable position used in bedrest studies to maintain the physiological effects of microgravity [23]. The total out-of-bath supine time during DI was 9.7 ± 1.3h. From DI-1 to DI-4, the out-of-bath time was 1.1 ± 0.6 h/day. On DI-5, the out-of-bath time was 5.3 ± 1.1 h to carry out Magnetic Resonance Imaging. Otherwise, during DI, subjects remained immersed in a supine position and were continuously monitored by video camera. Body weight, blood pressure, heart rate and tympanic temperature were measured daily. Participants in the Cuffs group wore the thigh cuffs from 10:00 (just before DI initiation) to 18:00 at DI-1 and from 8:00 to 18:00 from DI-2 to DI-5. Thigh cuffs are elastic strips, adapted to each subject to obtain the same effects on lower limb venous distensibility with a counterpressure of about 30 mmHg. For each participant, cuff adjustment was determined by calf plethysmography, performed in the supine position at DI-2.

### 2.5. Stool Collection and Metagenomic Analysis

Stool samples were collected from all participants without any constraint before DI initiation (DI-0) and at DI-5. However, gut microbiota composition changes before and after DI were assessed in 14 participants because participants E, M, H, and R did not provide stool samples at DI-0 and DI-5. Stool samples were stored at −80 °C until analysis.

#### 2.5.1. DNA Extraction from Feces

Total DNA was extracted from 0.1 g of fecal material using the G’NOME^®^ kit (BIO 10, MPBio, La Jolla, CA, USA) with modifications [24]. Fecal samples were homogenized in the supplied cell suspension solution. Cell lysis/denaturing solution was then added and samples were incubated at 55 °C for 2 h. To improve cell lysis, 0.1 mm-diameter silica beads (750 μL) were added, and samples were mixed at maximum speed in a Fast-Prep (MPBio, La Jolla, CA, USA) for 4 min. Polyvinylpolypyrrolidone (PVPP, 15 mg) was added to ensure removal of polyphenol that could inhibit the quantitative PCR (qPCR) assays. Samples were vortexed and centrifuged at 20,000× *g* for 3 min, and supernatants were recovered. Pellets were washed with 400 μL of TENP (50 mM Tris (pH 8), 20 mM EDTA (pH 8), 100 mM NaCl, 1% PVPP) and centrifuged at 20,000× *g* for 3 min. After another washing step, supernatants were pooled. DNA was precipitated by addition of one volume of isopropanol, incubated at −20 °C for 20 min, and centrifugated at 20,000× *g* for 10 min. Pellets were resuspended in 400 μL of distilled water with 100 μL of salt-out mixture, and incubated at 4 °C for 10 min. After spinning at maximum speed for 10 min, DNA-containing supernatants were transferred to clean 1.5-mL microcentrifuge tubes. DNA was precipitated with two volumes of 100% ethanol at room temperature for 5 min followed by centrifugation at 16,000× *g* for 5 min. DNA was resuspended in 150 μL of TE buffer and stored at −20°C.

#### 2.5.2. Evaluation of Total Bacteria by Real-Time qPCR Analysis of Bacterial 16 s rRNA Genes

The total bacteria present in the fecal samples of each participant was quantified by real-time qPCR using the universal primers F-bact1369 CGGTGAATACGTTCCCGG and R-prok1492 TACGGCTACCTTGTTACGACTT to target 16 S rRNA genes (“all bacteria” analysis) [24] and the StepOnePlus Real-Time PCR System (Applied Biosystems, Courtaboeuf, France). Each mixture contained 10 μL of Mastermix (PowerUpSybrGreen Master Mix, ThermoFisher Scientific, Courtaboeuf, France), 500 nM of forward and reverse primers, 5 μL of diluted cDNA template, and water to a final volume of 15 μL. All qPCR assays were performed in duplicate with the following cycling conditions: 50 °C for 2 min, then 95 °C for 2 min followed by 40 cycles of 95 °C for 3 s and 60 °C for 30 s, with a final melting step to improve the amplification specificity. For quantification, the Escherichia coli DNA standard curve was generated by plotting the threshold cycles (Ct) vs. bacterial quantity. The lower limit of detection for bacterial enumeration with good precision is 10^6^ bacteria per gram of stool.

#### 2.5.3. Phylum Abundance Quantification by Real-Time qPCR

Specific phyla were quantified using probes specific of the three main microbiota phyla, Firmicutes*^P^* (934F-Firm-5′-GGAGYATGTGGTTTAATTCGAAGCA-3′ and 1060-FirmR-5′-AGCTGACGACAACCATGCAC-3′), Bacteroidetes*^P^* (MIBF-5′-GGCGACCGGCGCACGGG-3′ and MIBR-5′-GRCCTTCCTCTCAGAACCC-3′), Proteobacteria*^P^* (ProteoF-5′-GCTCGTGTTGTGAAATGTTGG-3′ and ProteoR-5′-CGTAAGGGCCATGATGACTTG-3′), with the “all bacteria” protocol. The total number of bacteria was inferred from averaged standard curves as previously described [25,26]. For the quantification of Firmicutes^*P*^; Bacteroidetes^*P*^, and Proteobacteria^*P*^, standard curves were generated from serial dilutions of a known concentration of genomic DNA from *Lactobacillus acidophilus*, *Bacteroides fragilis* and *E. coli*, respectively.

#### 2.5.4. Microbiota Composition Analysis by Sequencing

The V3-V4 region of the bacterial 16S rRNA genes was amplified using the bacterial primers 343F (5′-CTT TCC CTA CAC GAC GCT CTT CCG ATC TAC GGR AGG CAG CAG-3′) and 784R (5′-GGA GTT CAG ACG TGT GCT CTT CCG ATC TTA CCA GGG TAT CTA ATC CT-3′), modified to add adaptors during the PCR amplification, the MolTaq 16S DNA polymerase and the corresponding master mix (Molzym GmbH and Co.KG, Bremen, Germany). The PCR mix contained 10 ng of DNA, 1 µL of dNTPs (10 mM), 1.25 µL each of forward and reverse primer (20 µM), and 0.5 µL of Taq polymerase in a total volume of 50 µL. The cycling program was: 94 °C for 3 min, followed by 40 cycles at 94 °C for 15 s, 60 °C for 30 s, 72 °C for 60 s, and a final extension at 72 °C or 5 min. Sequencing was performed with the MiSeq technology (Illumina) at the Genopole Toulouse Midi-Pyrenees (GeT) genomics facility (http://get.genotoul.fr/; accessed on 25 October 2021).

#### 2.5.5. Metagenomic Analysis

Sequencing data were demultiplexed at the GeT platform. The Galaxy-supported pipeline FROGS (Find, Rapidly, Otus with Galaxy Solution) was used to analyze the obtained sequences and produce abundance tables of Operational Taxonomic Units (OTUs) and their taxonomic affiliation [27]. The most abundant sequences of each OTU were then affiliated with blastn against the Silva v128 database [28]. Abundance tables and taxonomy files were manually imported into RStudio (v1.2.1335). Analyses were performed with the Phyloseq 1.28.0 [29] and ggplot2 [30] packages and custom scripts. Samples were rarefied to even sampling depths before computing the α diversity (Observed richness, Chao1, Shannon and InvSimpson) and β diversity (Jaccard, Bray-Curtis, UniFrac) indices. Principal Coordinate Analysis (PCoA) was also performed on dissimilarity matrices to obtain a two-dimensional representation of the samples. Alpha diversity data were compared using the paired *t*-test when the assumptions of normality and/or equal variance were met, or the Wilcoxon rank sum test. Beta diversity data were compared with permutational multivariate ANOVA (PERMANOVA) tests using 9999 random permutations and a significance level of 0.01. The relative abundances of phyla were compared using the paired *t*-test. Default parameters were used for picrust2 (except for the NSTI cut-off> set to 1) and we examined the MetaCyc ontology predictions [31].

### 2.6. Short-Chain Fatty Acid Analysis

SCFA analysis was carried out as described previously [32] using stool samples stored at −80 °C. Thawed samples were water-extracted and proteins were precipitated with phosphotungstic acid. SCFA analysis was performed using 0.1 μL of supernatant fraction and a gas-liquid chromatograph (CP7580; Agilent, Les Ulis, France) equipped with a split/splitless injector, a flame-ionization detector, and a capillary column (15 m × 0.53 mm, 0.5 μm) impregnated with SP 1000 (FSCAP Nukol; Supelco, Saint-Quentin-Fallavier, France). The carrier gas (H_2_) flow rate was 10 mL/min, and the inlet, column and detector temperatures were 200 °C, 100 °C and 240 °C, respectively. 2-Ethylbutyrate was used as internal standard. Samples were analyzed in duplicate. Data were collected and peaks integrated using the Turbochrom v 6 software (Perkin Elmer, Courtaboeuf, France).

### 2.7. Participant Flow and Statistics

**Figure 1:**
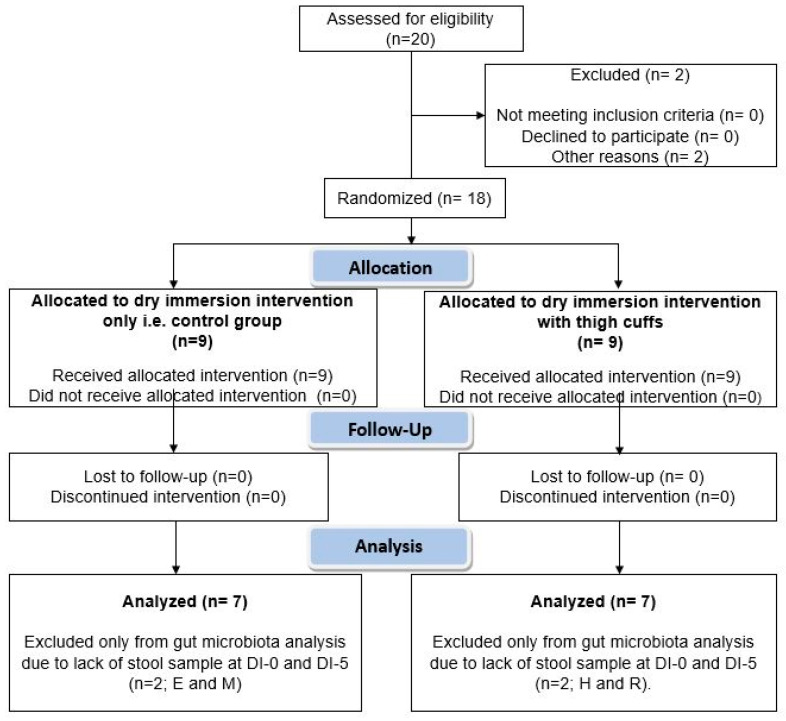
Participants’ flow chart. Twenty men were recruited to undergo dry immersion for 5 days. Before randomization, two participants were excluded. The remaining 18 men were divided in two groups: Control group (*n* = 9) and Cuffs group (with thigh cuffs; *n* = 9). Stool samples were collected at DI-0 and DI-5 for gut microbiota analysis. DI-0 = before Dry Immersion initiation; DI-5 = day 5 of Dry Immersion.

Figure 1 shows the participants’ flow. All data are presented as the mean ± SEM. The normality of each distribution and homogeneity of variance were assessed with the Kolmogorov–Smirnov and Fischer’s exact test, respectively. This clinical trial was originally designed to assess the effects of thigh cuffs on the hematological, cardiovascular and musculoskeletal system responses induced by 5 days of DI. Statistical significance was checked using a two-way ANOVA for repeated measures. As cuffs did not have any significant effects on the gut microbiota composition (Supplementary Figures S1 and S2), the Results and Discussion sections only focus on DI effects on gut microbiota. The paired *t*-test was used to compare directly DI-0 and DI-5. The Wilcoxon rank sum test was chosen when the normality and/or equal variance tests failed. For all statistical analyses, the significance level was set at 0.05. Data were analyzed using the statistical package GraphPad Prism version 6.02 for Windows (GraphPad Software, La Jolla, CA, USA).

## 3. Results

### 3.1. Dry Immersion-Induced Muscle Atrophy Despite a Controlled and Preserved Nutrient Intake

The DEXA analysis (Table 2) showed that the whole body and leg lean masses significantly decreased between BDC-4 and DI-5 (−2.5%, *p* < 0.001; and −2.9%, *p* < 0.001, respectively), confirming the induction of muscle atrophy by 5 days of DI. The fat mass percentage was unchanged in all participants (24.0 ± 2.9% at BDC-4 and 23.9 ± 3.0% at DI-5). The daily nutrient intake (carbohydrates, proteins, total fat and fatty acids, water, fibers, main minerals, and vitamins) of each participant was recorded (Table 3 and Table 4). The daily caloric intake was ~2625 kcal before DI and 2160 kcal during the DI period (not significantly different). No time-effect of DI was observed on the nutrient intake.

### 3.2. The Abundance of the Main Microbiota Phyla Is Not Affected by Dry Immersion

**Figure 2:**
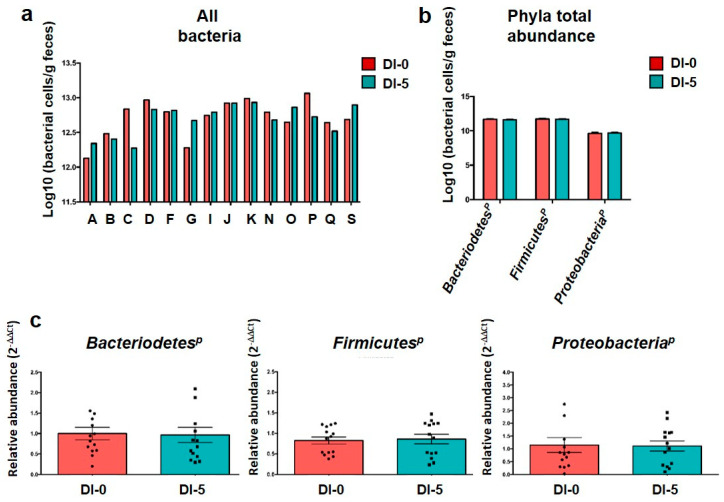
Gut microbiota composition analysis by qPCR. (**a**) “All bacteria” abundance evaluated by qPCR before (DI-0) and after 5 days of dry immersion (DI-5) in healthy men (*n* = 14). (**b**) Mean abundance of the three main gut microbiota phyla quantified by qPCR at DI-0 and DI-5 in healthy men (*n* = 14). (**c**) Mean abundance of each phylum by qPCR normalized to the “all bacteria” abundance at DI-0 and DI-5 in healthy men (*n* = 14). No significant difference between DI-0 and DI-5 for all panels (paired *t*-test); *p* = Phylum. Data are mean ± SEM.

To confirm the similar quantity and quality of DNA extraction between groups, an “all bacteria” qPCR analysis was performed (Figure 2a). As expected, overall, “all bacteria” quantity was comparable among subjects at DI-0 (12.71 ± 0.26 log of bacterial cells/g feces) and DI-5 (12.69 ± 0.22 log of bacterial cells/g feces), thus confirming the robustness of the extraction method. Moreover, the qPCR analysis suggested that the abundance of the three main phyla was not changed between DI-0 and DI-5: Bacteroidetes^*P*^ (11.66 ± 0.10 log10 vs. 11.62 ± 0.08 log10), Firmicutes^*P*^ (11.72 ± 0.10 log10 vs. 11.68 ± 0.09 log10) and Proteobacteria^*P*^ (9.62 ± 0.18 log10 vs. 9.67 ± 0.14 log10) (Figure 2b). Due to its robustness, the “all bacteria” quantification was then used as a baseline control for phylum quantification by qPCR (“housekeeping” all-bacteria). Statistical analysis of each phylum abundance relative to the “all bacteria” abundance, expressed as 2^−ΔΔCt^, showed no difference before (DI-0) and DI-5 (Figure 2c). This was confirmed by the metagenomic analysis that did not show any variation in the abundance of these three main phyla and of Actinobacteria (see below).

### 3.3. Dry Immersion Does Not Significantly Affect α and β Diversity

**Figure 3:**
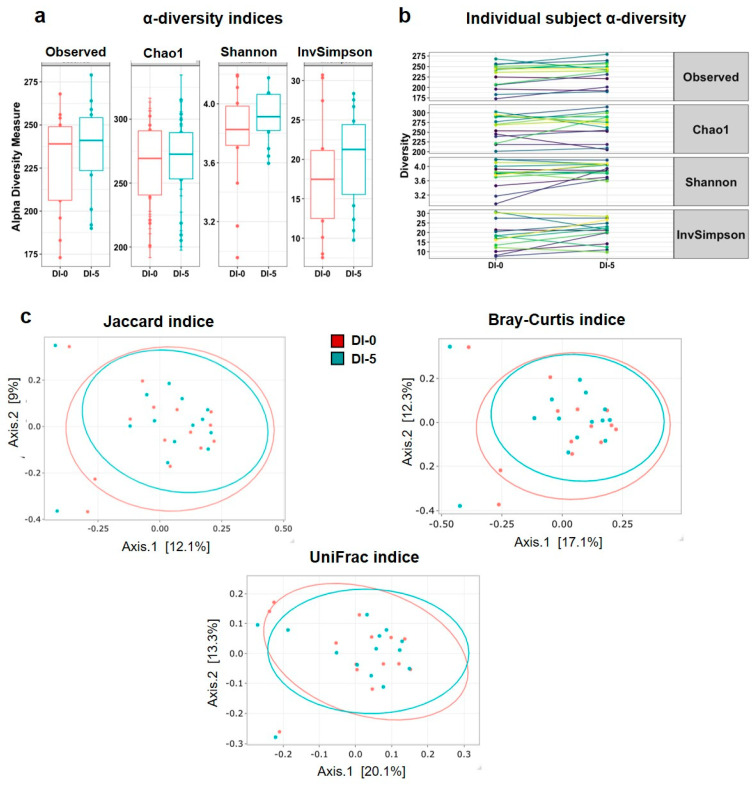
α and β diversity indices before (DI-0) and after 5 days of Dry Immersion (DI-5) in healthy men (*n* = 14). (**a**) α-diversity evaluated with the Observed, Chao1, Shannon and InvSimpson indices. The paired *t*-test did not find any significant difference between time points. (**b**) Individual α-diversity evaluated with the Observed, Chao1, Shannon and InvSimpson indices. (**c**) β-diversity analysis using the Jaccard (*p* = 0.998), Bray–Curtis (*p* = 0.997) and UniFrac (*p* = 0.999) indices indicated no difference between DI-0 and DI-5 in microbial OTU absence/presence, abundance, or phylogeny (PERMANOVA analysis). Data are mean ± SEM.

To measure the overall microbiota changes after DI, the α and β diversity of the taxonomic profiles, obtained by 16S rRNA metagenomic sequencing of stool samples (*n* = 14 men), were compared before (DI-0) and after DI (DI-5). This analysis showed that α diversity (Observed, Chao1, Shannon and InvSimpson indices) was not modified by DI (Figure 3a). Moreover, the individual plots showed similar changes for all α diversity indices in all participants (Figure 3b), without significant differences between DI-0 and DI-5 (Observed: *p* = 0.166; Chao1: *p* = 0.984; Shannon: *p* = 0.121; InvSimpson: *p* = 0.348). These findings indicated the absence of DI effects on gut microbiota richness and evenness. Similarly, the PCoA plots (Figure 3c) established from dissimilarity matrices to explain the β diversity using the Jaccard (*p* = 0.998), Bray–Curtis (*p* = 0.997) and UniFrac (*p* = 0.999) indices did not detect any difference between DI-0 and DI-5. These results revealed the absence of DI effects on OTU diversity.

### 3.4. DI Affects OTUs Associated with the Clostridiales Order and the Lachnospiraceae Family with Impacts on Bacterial Functional Profiles Linked to Anaerobic Glycolysis

**Figure 4:**
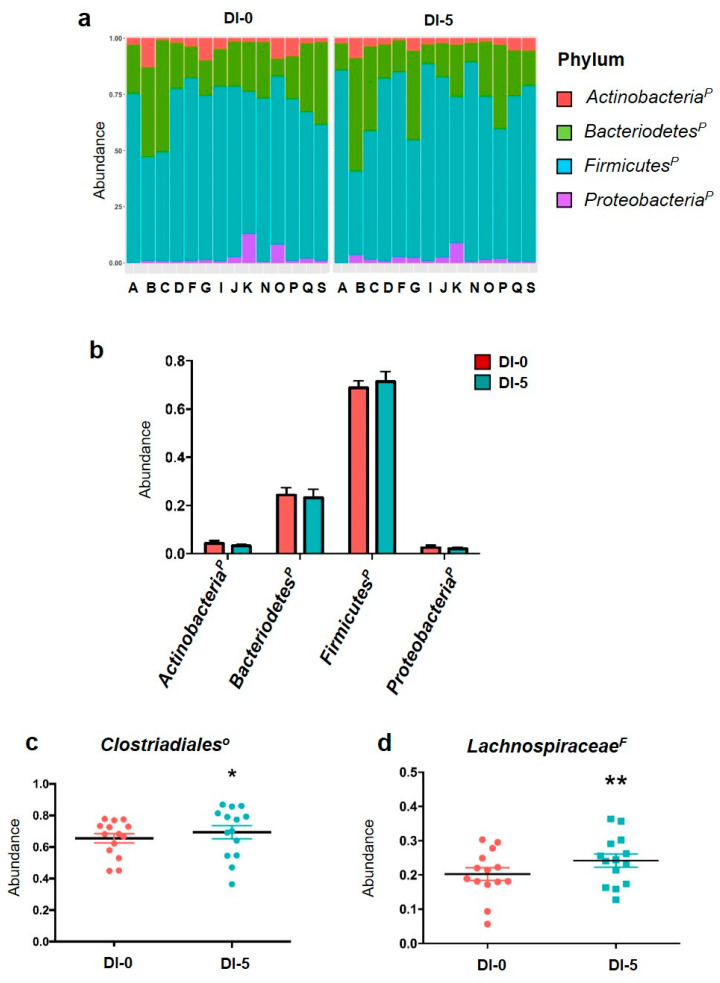
Abundance of phyla by 16S rRNA sequencing. (**a**) Individual abundance of the four main gut microbiota phyla in humans before (DI-0) and after 5 days of dry immersion (DI-5) in healthy men (*n* = 14). (**b**) Comparison (*t*-test) of the abundance of these four phyla at DI-0 and DI5 in healthy men (*n* = 14). (**c**) Clostridiales order abundance is significantly increased by DI. (**d)** Lachnospiraceae family abundance is increased by 5 days of dry immersion. *p* = Phylum; O = Order; F = Family; * *p* < 0.05; ** *p* < 0.01 (paired *t*-test). Data are mean ± SEM.

The metagenomic analysis gave similar results to the qPCR analysis for the individual and global phylum abundances (Figure 4a,b). The abundance of the four main phyla was not modified by 5 days of DI (Figure 4b). The total abundances of OTUs associated with orders belonging to the four main phyla for each participant are in Figure S3. The metagenomic analysis of the 32 families and 44 associated genera showed that 5 days of DI increased OTUs related to the Clostridiales order (Firmicutes family) from 65.5% up to 69.3% (*p* = 0.015; Figure 4c). Moreover, the Lachnospiraceae family (Firmicutes phylum) relative abundance was increased by DI (0.202 ± 0.01 at DI-0 vs. 0.241 ± 0.01 at DI-5; *p* < 0.01 (Figure 4d). The functional metagenomic contents inferred using PICRUSt2 analysis were examined to better understand how the bacterial functional profiles differed between DI-0 and DI-5. The MetaCyc ontology predictions showed that three pathways were significantly different using the Student *t* test: the homolactic fermentation (ANAEROFRUCAT-PWY; *n* = 14; DI-0 = 6350.5 ± 262.8 vs. DI-5 = 5289.1 ± 377.1; *p*-value = 0.035) and the glycolysis (GLYCOLYSIS; *n* = 14; DI-0 = 7399.8 ± 254.5 vs. DI-5 = 6300.0 ± 373.5; *p*-value = 0.027) in both pathways decreased in DI-5 vs. DI-0. Concerning the thiazole component of thiamine diphosphate biosynthesis I pathway (PWY-6892; *n* = 14; DI-0 = 5435.8 ± 160.8 vs. DI-5 = 6343.8 ± 272.6; *p*-value = 0.010), we highlighted an increase in DI-5 vs. DI-0.

### 3.5. Propionate Production Is Decreased by 5 Days of Dry Immersion

**Figure 5:**
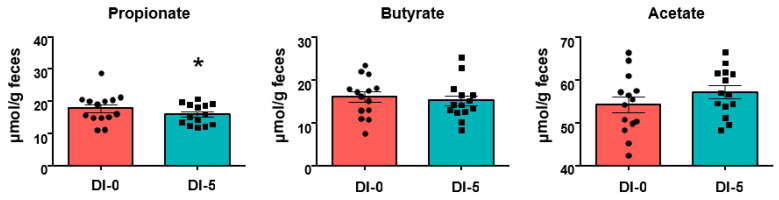
SCFA quantification in stool samples (*n* = 14) collected before (DI-0) and after 5 days of dry immersion (DI-5). * *p* < 0.05 (paired Student-*t* test). Data are mean ± SEM.

SCFAs quantification in stool samples highlighted a significant decrease of propionate concentration at DI-5 compared with DI-0 (17.8 ± 1.2 μmol/g feces at DI-0 vs. 16.0 ± 0.8 μmol/g feces at DI-5, *p* < 0.05) (Figure 5). DI did not have any effect on butyrate and acetate.

## 4. Discussion

Our results showed that a short period of severe hypoactivity, which is enough to induce skeletal muscle atrophy in healthy humans, increased the OTUs associated with the Clostridiales order and the Lachnospiraceae family, which belong to the Firmicutes phylum, without any effect on α- and β-diversity indices. Moreover, propionate, a SCFA metabolized by skeletal muscle, was significantly reduced in the stool samples collected after the hypoactivity period.

Head-down bedrest and more recently DI have been validated as reliable ground-based models to study the physiological effects of hypoactivity in humans and have been successfully used to describe and evaluate the muscle changes associated with reduced activity [19,23,33,34,35,36]. However, DI reproduces the effects of hypoactivity on the musculoskeletal system more rapidly than the head-down bedrest approach [19,35,36]. The negative impact of hypoactivity on the skeletal muscle mass is well-documented in healthy subjects during immobilization or with a sedentary lifestyle, during aging, in chronic diseases and also in microgravity. It is explained mainly by the loss of muscle mass and myofiber atrophy, induced by the deregulation of signaling pathways that regulate the protein balance in muscles (i.e., proteolysis, apoptosis) [34,37]. Studies in animal models and humans have shown that the decrease in muscle mass is exponential and involves major changes in the first days [38,39,40]. In the present study, DEXA analysis showed that 5 days of DI had a significant effect on leg lean mass (−2.5%), confirming the induction of skeletal muscle atrophy by DI. This result is in accordance with the significant reduction of myofiber cross-sectional area observed after 3 days of DI [19]. As the participants’ nutrient/calorie intake was strictly monitored and did not change significantly during the intervention, our study highlights a direct effect of hypoactivity on skeletal muscle atrophy, independent of the energy intake.

The current literature supports the notion that the gut microbiota composition is modulated by physical exercise and diet, with a functional “gut–skeletal muscle axis” [41] in healthy, athletes and in older adults. Conversely, the impact of hypoactivity on intestine bacteria remains largely unknown, and the available data come mainly from microgravity studies. The present work in healthy men, using DI, provides some original insights into this issue.

First, DI did not induce any significant global composition change at the phylum level, as already reported in human and murine spaceflight studies [16,32] despite the significant effect on muscle atrophy. Similarly, α and β diversities were not changed by 5 days of DI, partially in agreement with data from microgravity studies. Indeed, Voorhies and colleagues did not find any change in α diversity in humans after a spaceflight mission of 6 months to 1 year [16]. Conversely, β diversity was transiently modified during the spaceflight mission, but without any significant difference between the pre- and post-mission values. Similarly, Ritchie and colleagues did not observe any change in α diversity after 13 days of spaceflight in a murine model, but reported a different clustering by PCoA (β diversity) between the ground (control) and flight groups [42]. The absence of β diversity changes after DI in our study could confirm the stability of the human gut microflora [43].

On the other hand, at the lower taxonomy levels, DI induced significant alterations in OTUs assigned to the Clostridiales order and the Lachnospiraceae family. DI induced an increase in OTUs from the Clostridiales order, which represents ~70% of the Firmicutes phylum. Similarly, OTUs associated with the Clostridiales order were increased (+60%) in rats after 13 days of spaceflight [32]. The Lachnospiraceae*^F^* family is a phylogenetically and morphologically heterogeneous taxon belonging to the Clostridium cluster XIVa of the Firmicutes phylum [44]. In their hosts, this family of anaerobic bacteria produces SCFAs, converts primary to secondary bile acids, and promotes resistance against colonization by intestinal pathogens. In our study, the significant higher abundance of *Lachnospiraceae*-associated OTUs could be interpreted as a positive adaptation to hypoactivity because its reduction has been previously associated with negative health implications [44]. Moreover, the introduction of this family as probiotics increases the immune resistance against pathogenic bacteria, such as *Clostridium difficile* [45]. Nevertheless, Sorbara et al. highlighted the inter- and intra-species diversity of commensal bacterial species belonging to the *Lachnospiraceae* family, an important finding because a family member (*Ruminococcus gnavus*) has been implicated in Crohn’s disease pathogenesis [46]. These results suggest the potential impact of hypoactivity-related microbiota changes on systemic inflammation and immune components [17]. Interestingly, an increase of *Lachnospiraceae* OTUs was observed also in the hindlimb unloading mouse model of hypoactivity [47]. Similarly, various genera and species belonging to the *Lachnospiraceae* family were increased in mice after 37 days in the International Space Station [29]. These data suggest that the *Lachnospiraceae* family is particularly sensitive to hypoactivity and might play a key role in the hypoactivity–gut microbiota axis. Moreover, the functional metagenomic contents inferred by using PICRUSt2 analysis suggested bacterial anaerobic glycolysis pathway impairment. However, this interesting prediction would need further investigations in the future to delineate possible consequences on the host’s metabolism.

Finally, we found that the SCFA propionate was decreased in feces after 5 days of DI, suggesting that the hypoactivity period hampered its production in the intestine. Our results on the positive effect of DI positive *Clostridiales* and *Lachnospiraceae* abundance suggest a limited role of these bacteria in its production compared with other SCFA producers, such as *Faecalibacterium*, *Succinivibrio*, and *Butyricimonas.* Interestingly, SCFAs are the most studied putative mediators of the gut microbiota effect on skeletal muscle metabolism and function [41,48,49]. Thus, the propionate decrease after DI raises questions about its impact on muscle function because propionate metabolization is important for ATP production, and rectal inoculation of this SCFA increases treadmill running time in mice [14]. As DI was only for 5 days, we cannot exclude more important effects of a longer hypoactivity period on the gut microbiota composition, particularly on SCFA producers, and consequently on skeletal muscle metabolism and function [49]. Thus, the negative impact of hypoactivity on skeletal muscle could be partly explained by gut microbiota alterations and the associated SCFA-mediated metabolic effects. In this context, the head-down bedrest approach used to explore the effects of weightlessness and hypoactivity in humans for longer periods (up to several months) is a complementary and interesting strategy to further study the hypoactivity–gut microbiota–skeletal muscle axis.

Collectively, our findings suggest that the human gut microbiota, a major determinant of the host’s health, is sensitive to hypoactivity and justifies more research on this topic because sedentary lifestyles are widespread and have many negative health and socio-economic consequences. Future studies should investigate the mechanisms underlining the gut microbiota role in hypoactivity and disuse and the impact of hypoactivity on the “gut–skeletal muscle axis”. The integration of all these data might lead to the identification of key microbial taxa and microbial markers of hypoactivity that could be used to propose nutritional recommendations for targeted microbiota-based therapeutic approaches with the aim of limiting the negative impact of hypoactivity on the host’s health.

## Figures and Tables

**Table 1:** Baseline characteristics at day 2 before DI initiation (BDC-2).

	CTL (*n* = 9)	CUFFS (*n* = 9)	ALL (*n* = 18)
Age (years)	33.4 ± 7.1	33.8 ± 3.7	33.6 ± 5.5
Height (cm)	176 ± 6	180 ± 4	178 ± 6
Weight (kg)	73.9 ± 7.5	74.3 ± 8.8	74.4 ± 8.0
BMI (kg/m^2^)	23.9 ± 1.7	22.7 ± 1.8	23.5 ± 1.9
VO_2_max (ml/min/kg)	46.5 ± 8.1	46.9 ± 5.8	46.7 ± 6.9
Morning HR (bpm)	57 ± 6	58 ± 8	58 ± 7
Morning T (°C)	36.4 ± 0.3	36.4 ± 0.5	36.4 ± 0.4
Morning SBP (mmHg)	115 ± 11	117 ± 10	116 ± 10
Morning DBP (mmHg)	68 ± 5	68 ± 9	68 ± 7

Data are the mean ± SD. BDC = Baseline Data Collection. BMI = Body Mass Index; VO_2max_ = Maximal O_2_ Consumption; HR = Heart rate; T = Temperature; SPB = Systolic Blood Pressure; DPB = Diastolic Blood Pressure; DI = Dry Immersion; CTL = control group (no thigh cuffs); CUFFS = with thigh cuffs; ALL = whole samples. Statistical significance was checked using an unpaired T-test.

**Table 2:** Impact of 5 days of dry immersion on whole body and leg (right and left) lean mass.

	CTL (*n* = 9)	CUFFS (*n* = 9)	ALL (*n* = 18)
Whole body lean mass (kg)			
BDC-4	55.5 ± 4.7	55.8 ± 6.7	55.6 ± 5.6
DI-5	54.2 ± 4.5 ***	54.3 ± 6.5 ***	54.2 ± 5.4 ***
Leg lean mass (kg)			
BDC-4	18.3 ± 2.5	18.4 ± 1.9	18.3 ± 2.2
DI-5	17.6 ± 2.1 ***	17.7 ± 1.7 ***	17.7 ± 1.9 ***

Data are the mean ± SD. DI = Dry Immersion; BDC = Baseline Data Collection; CTL = control group; CUFFS = group with cuffs; ALL = whole samples. Only the time effect (DI-5 vs. BDC-4) was significant (*** *p* < 0.001), whereas no group or interaction effects were observed (two-way ANOVA).

**Table 3:** Daily Nutrient Intake.

Time	Precribed Energy (Kcal)		Energy (kcal)		Carbohydrates (g)		Proteins (g)		Total Fat (g)			
	Mean	SD	Mean	SD	Mean	SD	Mean	SD	Mean	SD		
**BDC-4**	2624.8	241.7	2625.1	241.3	304.0	28.4	81.5	8.0	101.2	9.3		
**BDC-3**	2624.8	241.7	2624.8	241.6	322.6	30.9	85.1	8.4	103.9	9.8		
**BDC-2**	2624.8	241.7	2624.6	241.5	324.5	32.3	86.7	8.4	102.7	9.3		
**BDC-1**	2624.8	241.7	2624.6	241.2	322.8	34.4	87.4	8.9	101.3	8.8		
**DI-1**	2160.1	205.7	2152.0	210.	252.8	27.6	80.6	6.3	82.3	8.7		
**DI-2**	2160.1	205.7	2160.5	205.2	250.9	24.3	78.3	7.4	86.3	9.0		
**DI-3**	2160.1	205.7	2160.4	205.7	255.5	23.0	85.7	9.7	83.5	8.9		
**DI-4**	2160.1	205.7	2160.2	205.5	252.7	24.6	82.4	7.8	83.6	8.1		
**DI-5**	2160.1	205.7	2160.5	205.5	237.5	25.1	75.3	7.9	81.6	8.1		
**R+0**	2658.8	253.1	2658.6	253.0	328.0	31.5	84.8	7.6	105.3	10.7		
**R+1**	2658.8	253.1	2658.9	253.0	325.9	30.7	85.8	8.0	102.8	10.7		
**Time**	**Saturated fatty acids (g)**		**Monounsaturated fatty acids (g)**		**Polyunsaturated fatty acids (g)**		**Total water (g)**		**Fibers (g)**			
	**Mean**	**SD**	**Mean**	**SD**	**Mean**	**SD**	**Mean**	**SD**	**Mean**	**SD**		
**BDC-4**	36.1	3.6	34.7	3.9	22.9	2.2	3422.6	530.2	39.2	3.3		
**BDC-3**	35.6	3.3	31.1	3.4	24.5	2.5	3462.5	485.0	35.0	2.8		
**BDC-2**	28.1	2.5	41.8	5.1	22.6	1.5	3585.2	450.0	35.7	3.4		
**BDC-1**	23.6	2.3	38.1	3.8	27.2	3.3	3490.4	532.8	40.1	2.8		
**DI-1**	17.8	2.1	31.1	4.5	24.8	2.6	3043.0	608.6	36.1	3.1		
**DI-2**	25.5	3.1	33.5	3.6	17.2	2.0	3307.3	492.8	32.1	2.2		
**DI-3**	24.0	2.7	31.7	3.8	19.1	1.9	3199.1	504.4	28.5	2.2		
**DI-4**	21.9	2.2	29.4	3.7	20.3	2.1	3287.9	465.1	33.3	2.9		
**DI-5**	30.3	2.9	27.2	3.9	18.0	1.8	3111.8	552.1	33.1	3.1		
**R+0**	35.0	4.0	32.0	3.6	25.5	2.9	4032.4	616.5	35.1	2.7		
**R+1**	22.4	2.3	41.3	5.8	29.3	3.0	3793.3	548.3	46.2	3.5		
**Time**	**Sodium** **(mg)**		**Chloride** **(mg)**		**Potassium** **(mg)**		**Calcium** **(mg)**		**Magnesium** **(mg)**		**Phosphorus** **(mg)**	
	**Mean**	**SD**	**Mean**	**SD**	**Mean**	**SD**	**Mean**	**SD**	**Mean**	**SD**	**Mean**	**SD**
**BDC-4**	3632.3	373.6	6656.0	674.7	3548.1	286.3	1360.5	112.5	353.3	33.1	1319.2	120.7
**BDC-3**	3726.3	349.3	7027.0	670.3	4974.4	427.3	1399.9	150.6	392.5	36.0	1620.3	169.3
**BDC-2**	3109.2	284.2	5308.4	460.8	3424.1	229.2	1231.7	74.5	419.5	31.9	1134.7	89.9
**BDC-1**	4006.2	424.4	7255.6	757.7	4480.5	284.8	1327.9	103.8	479.4	29.5	1364.8	113.2
**DI-1**	2392.5	208.0	4787.9	373.0	3440.3	211.0	1118.4	102.9	398.3	26.3	1396.3	107.7
**DI-2**	3034.6	308.5	5364.8	542.1	2986.5	186.7	1025.9	101.6	322.9	23.5	1260.0	112.6
**DI-3**	2837.7	266.5	4856.9	392.7	3101.4	132.9	1133.3	106.3	375.9	26.3	1071.1	106.0
**DI-4**	3620.7	331.0	6478.1	607.5	3690.0	329.5	1233.0	95.8	406.4	32.8	1247.1	109.9
**DI-5**	3036.4	280.8	5509.5	503.0	3286.5	274.2	1254.1	122.3	310.6	24.6	1198.9	102.4
**R+0**	3755.6	299.8	6953.9	578.0	4953.1	333.9	1407.2	146.0	408.7	33.8	1614.4	148.7
**R+1**	2706.0	197.8	5305.3	384.5	3934.8	283.6	1251.3	115.6	463.9	39.0	1511.9	142.2

Data are the mean ± SD; BDC-4: Baseline Data Collection, four days before DI; BDC-3: Baseline Data Collection, three days before DI; BDC-2 : Baseline Data Collection, two days before DI; BDC-1 : Baseline Data Collection, one day before DI; DI 1: first day of Dry Immersion; DI 2: second day of Dry Immersion; DI 3: third day of Dry Immersion; DI 4: fourth day of Dry Immersion; DI 5: fifth day of Dry Immersion; R + 0: first day of ambulatory recovery; R + 1: second day of ambulatory recovery; no time effect (two-way ANOVA for repeated measures).

**Table 4:** Daily Vitamin Intake.

Time	Vitamin A (µg_RE)		Vitamin K (µg)		Vitamin C (mg)		Niacin (vit PP) (mg)		Riboflavin (vit B-2) (mg)	
	Mean	SD	Mean	SD	Mean	SD	Mean	SD	Mean	SD
**BDC-4**	1736.2	162.2	301.8	29.2	406.0	45.0	30.9	3.1	2.3	0.2
**BDC-3**	911.7	123.8	520.7	51.5	191.7	18.8	43.1	4.6	2.1	0.2
**BDC-2**	2712.2	149.3	95.6	10.2	86.64	10.7	29.0	3.1	1.7	0.1
**BDC-1**	898.4	56.6	483.6	35.7	402.3	16.9	37.0	3.6	2.1	0.2
**DI-1**	1924.8	123.0	496.5	37.1	179.5	14.9	46.2	3.8	1.6	0.1
**DI-2**	826.1	48.4	487.5	47.6	170.2	13.0	31.4	2.8	2.0	0.1
**DI-3**	2397.6	169.1	74.4	5.8	71.2	6.6	28.5	3.3	1.5	0.1
**DI-4**	830.8	59.1	473.5	38.7	358.0	34.0	34.9	3.2	1.9	0.2
**DI-5**	1489.1	111.4	277.5	27.0	372.5	43.3	28.3	2.8	2.0	0.2
**R+0**	878.2	131.2	503.2	41.6	194.5	23.6	43.3	3.4	2.1	0.2
**R+1**	2162.4	219.0	507.9	51.2	210.8	19.1	46.9	4.6	1.9	0.2
**Time**	**Pantothenic Acid** **(vit B-5) (mg)**		**Thiamin** **(vit B-1) (mg)**		**Vitamin D** **(µg)**		**Biotin** **(vit H) (µg)**			
	**Mean**	**SD**	**Mean**	**SD**	**Mean**	**SD**	**Mean**	**SD**		
**BDC-4**	6.7	0.6	2.0	0.2	1.5	0.5	33.7	2.6		
**BDC-3**	8.0	0.8	2.0	0.2	3.1	0.5	57.0	5.9		
**BDC-2**	5.5	0.4	1.8	0.1	2.9	0.2	42.8	2.9		
**BDC-1**	6.6	0.5	2.3	0.2	1.5	0.2	45.4	3.6		
**DI-1**	5.8	0.4	1.8	0.1	2.9	0.3	50.2	3.9		
**DI-2**	5.2	0.3	1.3	0.1	1.8	0.3	42.6	2.9		
**DI-3**	5.0	0.4	1.6	0.1	2.7	0.3	37.6	2.8		
**DI-4**	6.0	0.5	2.1	0.2	1.4	0.1	36.1	3.3		
**DI-5**	6.0	0.5	1.6	0.1	1.1	0.1	28.5	3.0		
**R+0**	7.9	0.7	2.0	0.2	3.2	0.4	56.7	6.1		
**R+1**	6.4	0.5	2.2	0.2	3.0	0.3	57.4	4.6		
**Time**	**Vitamin E** **(mg)**		**Vitamin B-12** **(cobalamin) (µg)**		**Vitamin B-6** **(mg)**		**Folate** **(vit B9) (µg)**			
	**Mean**	**SD**	**Mean**	**SD**	**Mean**	**SD**	**Mean**	**SD**		
**BDC-4**	23.9	2.7	3.9	0.5	2.1	0.2	485.8	44.1		
**BDC-3**	25.7	2.6	4.3	0.6	2.4	0.2	542.2	56.4		
**BDC-2**	25.2	3.1	3.6	0.3	2.0	0.2	315.0	21.4		
**BDC-1**	28.3	3.1	3.0	0.4	3.3	0.2	487.8	28.0		
**DI-1**	30.9	3.3	4.1	0.4	2.5	0.2	517.3	46.5		
**DI-2**	16.4	1.7	3.5	0.4	2.0	0.1	383.0	21.1		
**DI-3**	19.4	2.1	3.6	0.4	1.8	0.1	268.9	17.1		
**DI-4**	20.3	2.1	3.1	0.3	2.8	0.3	419.0	33.3		
**DI-5**	19.3	2.1	4.0	0.5	1.8	0.1	442.3	44.5		
**R+0**	25.7	2.6	4.3	0.5	2.4	0.2	553.5	63.8		
**R+1**	37.8	4.0	3.8	0.5	2.8	0.2	633.9	49.1		

Data are the mean ± SD; BDC-4: Baseline Data Collection, four days before DI; BDC-3: Baseline Data Collection, three days before DI; BDC-2: Baseline Data Collection, two days before DI; BDC-1: Baseline Data Collection, one day before DI; DI 1: first day of Dry Immersion; DI 2: second day of Dry Immersion; DI 3: third day of Dry Immersion; DI 4: fourth day of Dry Immersion; DI 5: fifth day of Dry Immersion; R + 0: first day of ambulatory recovery; R + 1: second day of ambulatory recovery; no time effect (two-way ANOVA for repeated measures).

## Data Availability

Publicly available datasets were analyzed in this study. This data can be found here: https://data.inrae.fr/dataset.xhtml?persistentId=doi:10.15454/CGG7HW, accessed on 24 October 2021.

## Supplementary Materials

The following are available online at https://www.mdpi.com/article/10.3390/nu13113865/s1, Figure S1: Absence of thigh-cuffs effect on α-diversity gut microbiota before (DI-0) and after (DI-5) 5-days Dry 2 Immersion in healthy men, Figure S2: Absence of thigh-cuffs effect on β-diversity gut microbiota before (DI-0) and after (DI-5) 5-days of Dry 4 Immersion in healthy men, Figure S3: Graphical representations of OTUs affiliated to families belonging to Actinobacteria^*P*^, Bacteroidetes^*P*^, 6 Firmicutes^*P*^ and Proteobacteria^*P*^ before (DI-0) and after (DI-5) 5-days of Dry Immersion in healthy men.
