# Supplementary material for: Does Physical Inactivity Induce Significant Changes in Human Gut Microbiota? New Answers Using the Dry Immersion Hypoactivity Model
Source: Nutrients. 2021 Oct 29;13(11):3865. doi: 10.3390/nu13113865 (PMC8620432; doi:10.3390/nu13113865)
Supplement: Supplementary file 1 [file nutrients-13-03865-s001.zip › nutrients-1414680-supplementary.pdf]

Supplementary Figures:

Supplemental Fig S1. Absence of thigh-cuffs effect on α-diversity gut microbiota before (DI-0) and after (DI-5) 5-days Dry Immersion in healthy men; CTL = control group; CUFFS = group with cuffs.

Supplemental Fig S2. Absence of thigh-cuffs effect on β-diversity gut microbiota before (DI-0) and after (DI-5) 5-days of Dry Immersion in healthy men. ^{P} = phylum ; CTL = control group; CUFFS = group with cuffs.

Supplemental Fig S3. Graphical representations of OTUs affiliated to families belonging to Actinobacteria^{P}, Bacteroidetes^{P}, Firmicutes^{P} and Proteobacteria^{P} before (DI-0) and after (DI-5) 5-days of Dry Immersion in healthy men.

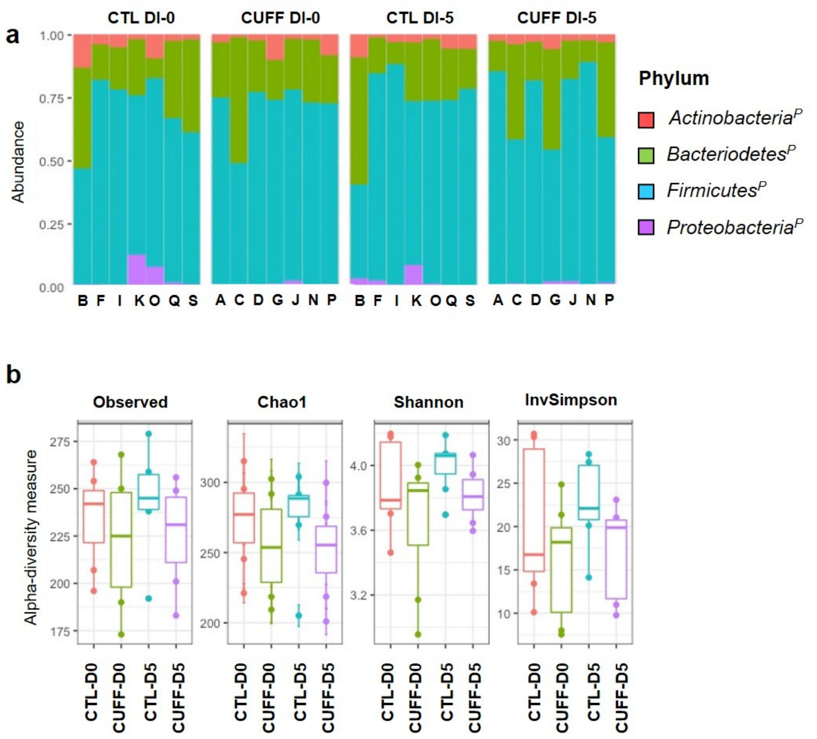

ANOVA significativity (a, b)				
	Actinobacteria^{P}	Bacteroidetes^{P}	Firmicutes^{P}	Proteobacteria^{P}
Group effect	0.052	0.343	0.204	0.128
Time effect	0.258	0.311	0.295	0.418
Interaction	0.314	0.914	0.627	0.126
	Observed	Chao1	Shannon	InvSimpson
Group effect	0.554	0.480	0.256	0.426
Time effect	0.905	0.522	0.084	0.426
Interaction	0.490	0.491	0.852	0.073

Supplemental Fig S1. Absence of thigh-cuffs effect on α-diversity gut microbiota before (DI-0) and after (DI-5) 5-days Dry Immersion in healthy men; CTL = control group; CUFFS = group with cuffs.

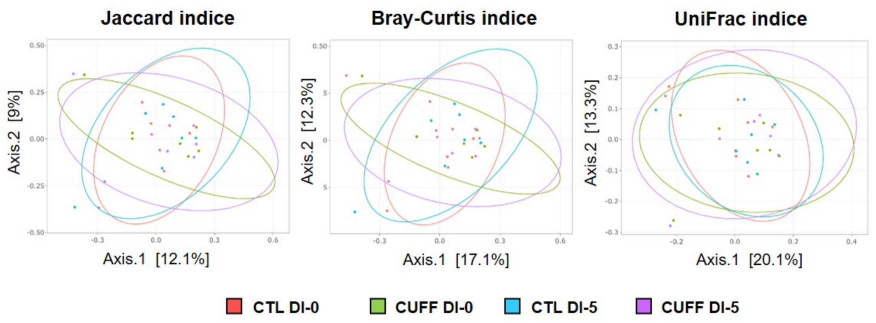

PERMANOVA significativity

	Jaccard	Bray-Curtis	UniFrac
**Group effect**	0.069	0.997	0.996
**Time effect**	p = 0.998	0.997	0.999
**Interaction**	0.998	0.111	0.999

Supplemental Fig S2. Absence of thigh-cuffs effect on β-diversity gut microbiota before (DI-0) and after (DI-5) 5-days of Dry Immersion in healthy men. P = phylum ; CTL = control group; CUFFS = group with cuffs.

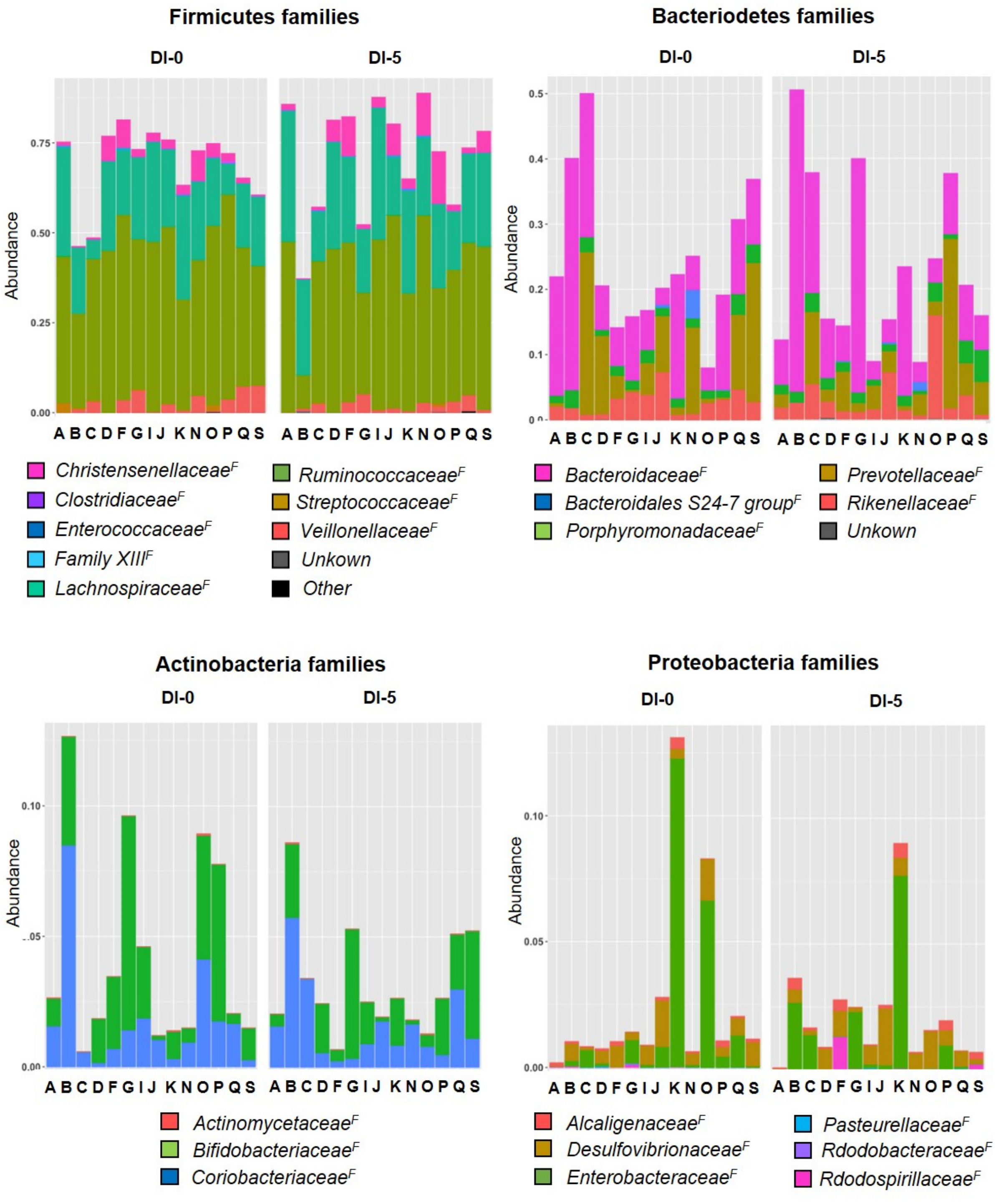

Supplemental Fig S3. Graphical representations of OTUs affiliated to families belonging to Actinobacteria[P], Bacteroidetes[P], Firmicutes[P] and Proteobacteria[P] before (DI-0) and after (DI-5) 5-days of Dry Immersion in healthy men.
